# Targeting Biomarkers of Proliferation and Inflammation (Ki67, p53, and COX-2) in Actinic Keratoses with Photodynamic Therapy

**DOI:** 10.3390/biomedicines13061487

**Published:** 2025-06-17

**Authors:** Justyna Ceryn, Aleksandra Lesiak, Magdalena Ciążyńska, Dorota Sobolewska-Sztychny, Marcin Noweta, Olga Stasikowska-Kanicka, Karol Ciążyński, Iris Zalaudek, Joanna Narbutt

**Affiliations:** 1 Department of Dermatology, Paediatric Dermatology and Dermatological Oncology, Medical University of Lodz, 90-217 Lodz, Poland; justyna.ceryn@stud.umed.lodz.pl (J.C.); lesiak_ola@interia.pl (A.L.); ciazynska.magdalena@gmail.com (M.C.); sobolewska.sztychny@gmail.com (D.S.-S.); m.noweta@wp.pl (M.N.); joanna.narbutt@umed.lodz.pl (J.N.); 2 International Doctoral School, Medical University of Lodz, 90-647 Lodz, Poland; justyna.ceryn@stud.umed.lodz.pl; 3 Laboratory of Autoinflammatory, Genetic and Rare Skin Disorders at Department of Dermatology, Pediatric Dermatology and Dermatological Oncology, Medical University of Lodz, 90-217 Lodz, Poland; lesiak_ola@interia.pl (A.L.); sobolewska.sztychny@gmail.com (D.S.-S.); joanna.narbutt@umed.lodz.pl (J.N.); 4 Chemotherapy Sub-Department and One-Day Chemotherapy Department, Specialist Oncological Hospital NU-MED sp. z o. o., 97-200 Tomaszów Mazowiecki, Poland; ciazynska.magdalena@gmail.com; 5 Department of Diagnostic Techniques in Pathomorphology, Medical University of Lodz, 92-213 Lodz, Poland; olga.stasikowska@umed.lodz.pl; 6 Institute of Applied Computer Science, Lodz University of Technology, 90-537 Lodz, Poland; karol.ciazynski@gmail.com; 7 Department of Dermatology, Maggiore Hospital, University of Trieste, 34127 Trieste, Italy; iris.zalaudek@asugi.sanita.fvg.it

**Keywords:** actinic keratosis, photodynamic therapy, Ki67, p53, COX-2

## Abstract

**Background**: Actinic keratoses (AKs) are common pre-neoplastic lesions that may progress to cutaneous squamous cell carcinoma (cSCC). Photodynamic therapy (PDT) is an effective field-directed treatment for AK, but its impact on key biomarkers remains unclear. This study evaluates the clinical, dermatoscopic, and immunohistochemical effects of PDT on AK, with a focus on proliferation (Ki67, p53) and inflammation (COX-2) markers, to assess its efficacy in delaying carcinogenesis. **Methods**: In our prospective one-center study, we enrolled 31 patients with AK, with no history of previous AK treatment. They underwent three PDT sessions at four-week intervals, with follow-up eight weeks after the final session. Clinical, dermatoscopic, and immunohistochemical analyses of Ki67, p53, and COX-2 expression were performed before and after treatment. **Results**: Clinically, 54.8% of patients achieved complete lesion clearance, with no residual severe AK lesions. Ki67 and p53 immunoexpression significantly decreased post-PDT (*p* < 0.05), confirming its antiproliferative effect. COX-2 expression also declined significantly (*p* < 0.05), supporting PDT’s anti-inflammatory role. However, COX-2 remained stable or increased in 35.48% of cases, possibly due to inflammation-induced regeneration. There is a positive correlation between the reduction in Ki67, p53, and COX-2 immunoexpression and the decrease in AK severity (both according to Olsen grade and dermatoscopic grade). **Conclusions**: PDT effectively reduces AK severity, proliferation, and inflammation markers, potentially delaying carcinogenesis. However, residual biomarker expression suggests that additional treatment sessions or combination therapies may be necessary for complete lesion clearance. Further studies are required to optimize PDT protocols.

## 1. Introduction

Actinic keratosis (AK) is a common benign intraepidermal dysplasia that may progress to cutaneous squamous cell carcinoma (cSCC). It affects approximately 25% of the adult population, particularly the elderly, with a prevalence of 4.6% in individuals aged 60–69 years and 14.57% in those over 80 years [1,2]. Clinically, AK presents as lesions characterized by hyperkeratosis and erythema. The primary cause of AK is prolonged exposure to ultraviolet (UV) light, with lesions typically occurring in sun-exposed areas such as the face, scalp, neck, and the dorsal surfaces of the hands [3]. Individuals with a low Fitzpatrick phototype (I or II) are more susceptible to developing AK [4]. The literature indicates that the risk of AK progressing to invasive SCC (iSCC) ranges from 0.025% to 16% per year [5,6] with a cumulative risk of approximately 40.7% within five years in patients with a history of cSCC [5,6,7]. Moreover, the presence of a previous cSCC within an AK lesion increases the risk of developing a second cSCC by 40.7% within five years [7]. In rare cases, AK may also transform into basal cell carcinoma (BCC) [8]. Signs suggesting the malignant transformation of AK into iSCC include lesion induration, bleeding, pain, and an increase in both thickness and diameter [9]. Additionally, AK lesions are associated with “field cancerization” (FC), a phenomenon in which an area of subclinical changes surrounds clinically visible AKs, displaying genetic alterations, among other clonal proliferations of p53- mutated fields, similar to those within the AK lesions [10]. FC also occurs in areas exposed to chronic UV radiation and is characterized by multifocal AKs, SCCs in situ, and SCCs [11]. Since AK is considered a precancerous condition, early treatment is crucial to prevent malignant progression.

AK treatment can be categorized as lesion-directed or field-directed. Lesion-directed treatments target individual AKs, while field-directed treatments address multiple, widespread, and subclinical AKs within FC [3,12]. Lesion-directed therapy is suitable for fewer than five AK lesions, mild to moderate photodamage, and a low risk of developing NMSC. Field-directed therapy is recommended for more than five AK lesions, severely UV-damaged skin, and a high risk of developing non-melanoma skin cancers (NMSCs). Both approaches include topical treatments (imiquimod, 5-fluorouracil, diclofenac) and physical methods (cryotherapy, photodynamic therapy (PDT), chemical peels, laser therapy, electrocoagulation, curettage).

One of the field-directed treatment options targeting the entire FC area is a PDT, which can be further categorized into conventional PDT (c-PDT) and daylight PDT (dl-PDT). According to the latest interdisciplinary European Guidelines for the treatment of AK (2024) [3] and the most recent guidelines published by the American Academy of Dermatology (AAD) in 2021 [12], all specialists agree on the high efficacy of PDT and have given it a strong recommendation. The effectiveness of PDT in AK treatment depends on various factors, including the available literature, the type of photosensitizer used, the number of PDT sessions, and, most importantly, the specific treatment protocol implemented. The most favorable protocol involves ALA–red light conventional PDT (c-PDT), which uses a 10% 5-aminolevulinic acid (ALA) gel (78 mg/g ALA nanoemulsion gel) as a photosensitizer, combined with a red-light source. According to the analysis conducted by the AAD Guidelines Work Group, the efficacy of this protocol in a single treatment session ranges from 77.1% to 89.1%. Moreover, it provides excellent cosmetic outcomes [12,13,14,15,16,17]. Furthermore, conventional nanoemulsion ALA-PDT has been shown to be superior to MAL (methyl aminolevulinate), another photosensitizer used in PDT, in the treatment of thin to moderately thick AK lesions on the face and scalp. Twelve weeks after one or two PDT sessions, the overall clearance rates were 90% for ALA-PDT versus 83% for MAL-PDT, with complete clearance rates of 78% and 64%, respectively [13]. Although the physical mechanism of PDT is well established, the exact molecular mechanisms underlying its effects on AK and FC lesions remain unclear.

In our study, we aimed to achieve the clinical resolution of AK lesions in our patients, inhibit the progression to cSCC, and additionally investigate selected molecular mechanisms involved in PDT treatment for AK. The specific objectives of our study included analyzing the impact of PDT on the expression of selected proliferation and inflammation biomarkers in AK.

Among the numerous biomarkers reported in the literature, we selected Ki67 and p53 as proliferation markers and COX-2 (cyclooxygenase-2) as an inflammation marker. For each patient, we performed clinical, dermoscopic, and histopathological evaluations of suspicious AK features both before and after PDT treatment. We then analyzed the expression of Ki67, p53, and COX-2 in skin samples from AK lesions and apparently healthy skin (control group) using immunohistochemistry.

Ki67 is an essential enzyme for DNA replication, playing a crucial role throughout the entire cell cycle, except during the M0 phase. It is widely used as a marker for evaluating cell proliferation. Typically, Ki67 is expressed in the basal layer of the epidermis and is strongly associated with tumor cell growth and proliferation. Several studies have confirmed its presence in both AK and FC lesions, with significantly higher concentrations in cSCC lesions (*p* < 0.01) [18]. Ki67 has also been studied in relation to various AK therapies; however, these aspects remain insufficiently explored.

The p53 protein plays a crucial role in DNA repair and apoptosis. Mutations in *TP53* represent the most common genetic alterations in human neoplasms [19]. While p53 is not expressed in healthy skin, it is present in sun-exposed skin, AK lesions (26–50%), and SCCs (12–64%) [20,21]. The role of p53 in skin malignancies is well-documented, with its expression frequently observed in sun-damaged skin. The *TP53* mutations are induced by ultraviolet radiation and found in 50–60% primary cSCCs and nearly 95% in metastatic cSCCs [22] whereas various sources report that genetic alterations in the *TP53* are present in approximately 30% to 75% of AK cases [23,24,25,26,27,28,29,30].

Furthermore, inflammation plays a critical role in the progression of AK and cSCC. The expression of COX-2, an isoform of cyclooxygenase, can be induced in the epidermis by various stimuli, including UV radiation—the primary trigger for AK development [31]. UV-induced COX-2 expression leads to elevated levels of prostaglandin E2 (PGE2), a major cyclooxygenase product implicated in the pathogenesis of NMSC. Additionally, COX-2 has been linked to several key processes, including angiogenesis, the inhibition of apoptosis, increased cell proliferation, enhanced invasiveness, immunosuppression, and the production of mutagens [32]. To our knowledge, this is the first study to investigate the effects of PDT on COX-2 expression in AK.

This study is the first to simultaneously evaluate Ki67, p53, and COX-2 expression in actinic keratoses before and after photodynamic therapy within a uniform treatment protocol. The inclusion of three mechanistically distinct markers allows for a multidimensional interpretation of therapeutic efficacy, offering new insights into the subclinical resolution of dysplasia and the biological normalization of photodamaged skin. This approach may contribute to the development of molecularly informed follow-up strategies and optimized treatment algorithms in field cancerization management.

## 2. Materials and Methods

### 2.1. Study Population

Adult patients from the Department of Dermatology, Paediatric Dermatology, and Oncology Clinic at the Medical University of Lodz, Poland, were included in this prospective single-center study. The study was approved by the Bioethics Committee of the Medical University of Lodz, and all patients provided written informed consent before participation. Several exclusion criteria were applied (outlined in Table 1), with the most significant being the absence of prior AK treatment, presence of active cancer (including other NMSCs, and malignant melanoma, MM), and chronic or intermittent use of nonsteroidal anti-inflammatory drugs (NSAIDs).

### 2.2. Study Design and Assessments (Clinical, Dermatoscopic, Histopathological, and Immunohistochemical Evaluation)

We scheduled three PDT sessions at four-week intervals. Subjects were assessed for AK lesions both clinically and via dermoscopy at baseline, before each PDT session, and again eight weeks after completing the treatment (final follow-up visit). Additionally, photographic and dermoscopic documentation of each lesion was performed at every visit. Lesions exhibiting characteristic scaling, keratotic patches, erythema, and a sandpaper-like texture were identified as AKs [33]. As for dermoscopic diagnosis, lesions exhibiting the following features were considered: erythema forming a pink-reddish vascular pseudonetwork surrounding hair follicles, yellowish-white scales, thin and wavy vessels surrounding the follicles, and follicular openings filled with keratotic plugs, collectively known as the “strawberry pattern” [34,35], and the “rosette” sign, a figure resembling a four-leaf clover, formed by four whitish points surrounding the follicular opening [36].

Additionally, we assessed the severity of AK using the clinical Olsen classification system, which includes grades 1, 2, and 3 [37]. Olsen’s classification system are presented in Table 2. Furthermore, we evaluated the severity of AK using the Dermatoscopic Severity Scale proposed by Zalaudek et al., with our slight modifications [35,38,39,40]. Dermatoscopically, we identified three common patterns associated with nonpigmented AK, classifying lesions into three grades, I, II, and III. Given the FC and the broader area affected by varying grades of AKs, we also introduced intermediate values, such as I/II and II/III, to better represent these transitional stages (see Table 2).

Regarding the PDT procedure, the identified AK lesions were degreased, cleared of scales and crusts, and then covered with a photosensitizing agent containing 5-aminolevulinic acid (5-ALA). Subsequently, the lesions were occluded with a non-absorbent, occlusive dressing for a period of 3 h. After this incubation period, the dressing was removed and the area was gently cleansed with a gauze pad soaked in 0.9% saline solution. The region was then exposed to red light (wavelength 630 ± 5 nm) using a lamp with the following parameters: energy dose of 37 J/cm^2^ and irradiance of 40 mW/cm^2^.

At baseline, prior to the first PDT session, we performed a punch skin biopsy (at least 4 mm in diameter) from both the AK lesion and apparently healthy skin (control group) to confirm the AK diagnosis and facilitate the planned immunohistochemistry analysis. At the final follow-up visit, another skin biopsy was taken from the previously treated AK lesions. If the selected AK for biopsy was no longer clinically visible at the final follow-up, a biopsy was still performed at the site where the lesion had been located (i.e., the regressed AK).

In accordance with established standards, tissue samples were immediately fixed in 10% buffered formalin after biopsy, processed using routine histopathological techniques, and stained with hematoxylin and eosin. The specimens were then evaluated according to current diagnostic criteria.

Immunohistochemical staining was performed using a standard protocol. Tissue sections (3 µm thick) were deparaffinized in xylene and rehydrated through a series of graded alcohols. Antigen retrieval was conducted by heating the sections in a microwave oven in TRS (Target Retrieval Solution, pH 9.0, Dako Denmark A/S, Glostrup, Denmark) for 30 min. Endogenous peroxidase activity was quenched with 0.3% hydrogen peroxide in methanol for 30 min. The sections were then washed with TBS and incubated for 30 min with primary antibodies against Ki67 (mouse monoclonal Ab; clone MIB-1; No kat M7240; dilution 1:100; Dako, Glostrup, Denmark), p53 (mouse monoclonal Ab; clone DO-7; No kat IR616, RTU; Dako Omnis), and COX-2 (rabbit polyclonal Ab; No kat RP-111; dilution 1:350; Diagnostic BioSystems, Pleasanton, CA, USA).

After washing, an adequate EnVision-HRP detection system (Dako, Carpinteria, CA, USA) was applied, with 3,3′-diaminobenzidine serving as the chromogen. Following counterstaining with Mayer’s hematoxylin, the slides were washed, dehydrated, cleared in xylene, and coverslipped. Negative controls underwent the same procedure, but with primary antibodies replaced by antibody diluent. The number of Ki67+ and p53+ cells (nuclear staining) was counted by two independent observers in 3 to 10 high-power fields (HPF), depending on the specimen size. Results were expressed as the mean number of immunopositive cells per HPF, with biomarker expression graded based on staining intensity and distribution. For p53 and Ki67, nuclear staining was scored as follows: 0 (<5% immunopositive cells); 1 (6–15%); 2 (16–30%); 3 (31–50%); 4 (>50% immunopositive cells). For the extent of Ki67 and p53 immunopositive cells in the epidermis, the grading was as follows: 0—no immunopositive cells; 1—<1/3 of the lower epidermal/lesion level; 2—1/3 to 2/3 of the epidermal/lesion level; 3—2/3 to the entire epidermis (as outlined in Table 3). For the statistical analysis, we used a Total Score, calculated as the sum of the grading of nuclear staining for Ki67 and p53, along with the extent of immunopositive cells for both markers. COX-2 immunoexpression (cytoplasmic staining) was assessed semi-quantitatively at 200× magnification, based on staining detectability. The mean grade was determined by averaging the scores assigned by two independent pathologists, rounding the arithmetic mean to the nearest integer. The grading for COX-2 was as follows: 0—no staining; 1—mild immunoexpression; 2—moderate immunoexpression; 3—strong immunoexpression (see Table 3).

### 2.3. Statistical Analysis

The primary objective of this analysis was to determine whether the biomarkers Ki67, p53, and COX-2 correlate with AK regression after PDT sessions and to assess whether their decreased immunoexpression in AK lesions following PDT is statistically significant (*p* < 0.05). For quantitative variables, data were analyzed using ANOVA or Student’s *t*-test. Differences were considered statistically significant for *p* < 0.05. Statistical analysis was performed using SPSS v20 (StatCorp, College Station, TX, USA).

## 3. Results

### 3.1. Study Group, Clinical, and Dermatoscopic Evaluation

**Figure 1:**
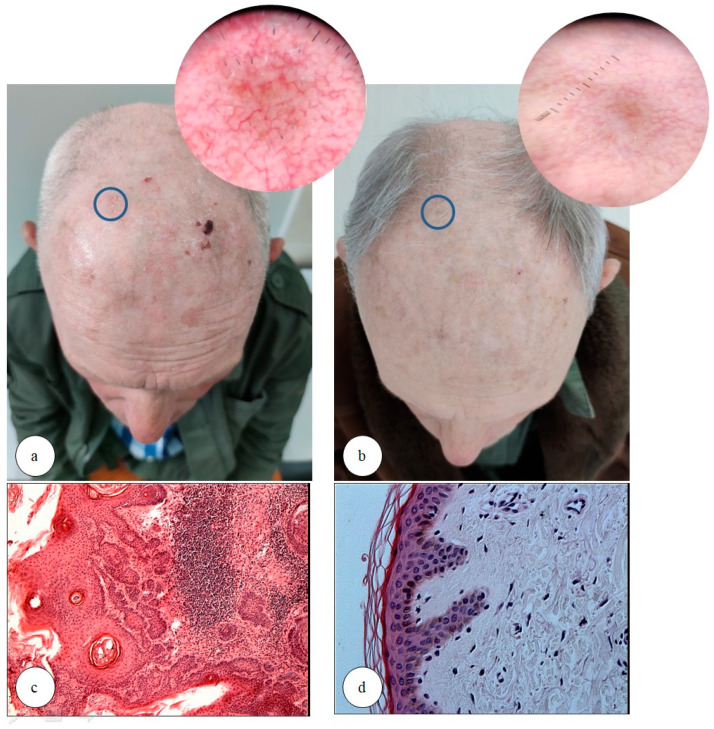
Clinical and dermatoscopic evaluation of actinic keratosis (AK) lesion before (**a**) and after (**b**) the photodynamic therapy (PDT); the blue circle shows the AK lesion before (**a**) and after treatment (**b**) and their dermoscopic images. Histological analysis of an AK lesion before (**c**) and after (**d**) PDT, showing morphological changes following treatment (hematoxylin and eosin (H&E) staining; magnification ×100 and ×200, respectively). Notable improvement in skin texture and a reduction in forehead wrinkles, as demonstrated in the clinical photographs (**a**,**b**), are consistent with the photorejuvenating effect of photodynamic therapy, which was observed in 16 of the 31 patients enrolled (51.61%). It is important to acknowledge that slight photographic variability, particularly related to differences in lighting conditions, may have contributed minor artifacts in the visual documentation.

Thirty-one adult patients with AK were enrolled in our study (Table 4 summarizes the patient characteristics). The patients enrolled in the study presented with AK lesions localized on the scalp (representing the majority of patients, 17; 54.84%), as well as on the face, forearm, and trunk (see Table 4). Each patient’s AK diagnosis was confirmed through histopathological examination of skin samples prior to PDT sessions. Clinical and dermatoscopic improvement was observed in all patients (31 subjects, 100%) following PDT (see Figure 1). Clinically, 17 patients (54.8%) achieved complete resolution of AK lesions, while 14 patients (45.2%) showed a reduction in AK severity to grade 1 (according to Olsen’s scale) after PDT. Notably, none of the patients exhibited grade 2 or 3 lesions post-treatment. Moreover, age was found to significantly influence the clinical response to PDT. Patients younger than 72.4 years exhibited a statistically superior therapeutic outcome compared to older individuals (see Table 5). In multivariate analysis, only the younger age of patients was an independent predisposing factor for better treatment response (according to Olsen scale). In contrast, sex was not a significant determinant of clinical response, as males and females responded equally well to PDT.

**Table 4:** Patients characteristics: demographic and clinical data of enrolled patients before treatment (*n* = 31).

Characteristic	*n* (%)
Age (mean = 72.4 ± 8.64; range 54–92)	
≥72.4	18 (60)
<72.4	13 (40)
Sex	
Male	24 (77.42)
Female	7 (22.58)
Anatomic location	
Scalp	17 (54.84)
Face	9 (29.03)
Forearm	3 (9.68)
Trunk	2 (6.45)
Fitzpatrick Skin Type	
I	3 (9.68)
II	26 (83.87)
III	2 (6.45)
AK clinical grade (Olsen’s Clasification System)	
1	
2	
3	
AK dermatoscopic grade (Dermatoscopic Severity Scale [35] with our modification)	
I	4 (12.9)
I/II	3 (9.68)
II	7 (25.81)
II/III	11 (35.48)
III	6 (19.35)

**Abbreviations:** AK, actinic keratosis.

**Table 5:** Clinical improvement after the treatment with photodynamic therapy. Total number of patients enrolled to the study, *n* = 31.

	Complete Resolution of Cutaneous Lesions		Olsen 1		
	*n*	%	*n*	%	
Age					*p* < 0.001 *
≥72.4	8	44	10	56	
<72.4	9	69	4	31	
Sex					*p* = 0.034
Male	12	50	12	50	
Female	5	71	2	29	

**Abbreviations:** * *p* < 0.05.

Dermatoscopic evaluation showed that eight patients (25.81%) achieved complete lesion clearance post-treatment. Additionally, 16 patients (51.61%) were reduced to grade I, and 7 patients (22.61%) to grade II. Notably, no patients exhibited grade III severity after PDT.

Adverse events (AEs) were reported by 18 patients (58.1%) during PDT sessions. The most common AEs, observed in 16 patients (51.6%), were mild local reactions, such as burning or pinching sensations during or after the procedure. These patients required special care, including pauses during irradiation, cooling air supply, reduced lamp power settings (energy dose of 37 J/cm^2^ but the irradiance was reduced by either 25% or 50%), or shortened exposure time (it was reduced by either 25% or 50%). Severe AEs occurred in two patients (6.45%), who developed burns in the treated area after the first PDT session. These cases required additional management, including topical and oral glucocorticosteroids and antihistamines, as well as an extended recovery period before the next session. Notably, these patients had the most severe AK lesions (Olsen 3 and high dermatoscopic grade), supporting the hypothesis that more severe AK lesions correlate with more intense local reactions following PDT. Moreover, these patients did not adhere to the recommended guidelines and exposed the treated AK lesions to UV light in the days following the procedure, which may have contributed to the severity of their reactions. On a positive note, 16 patients (51.61%) reported additional benefits, such as skin photorejuvenation, highlighting the favorable cosmetic outcomes of PDT and its potential to stimulate collagen synthesis [41,42,43]. In 15 cases (48.38%), post-treatment histopathological examination confirmed collagen thickening, which correlated with the clinical effects of skin photorejuvenation.

### 3.2. Ki67

Regarding Ki67 immunoexpression (1.16 ± 0.60), in apparently healthy skin (control group), immunoexpression was mild and restricted to the basal layer of the epidermis and skin appendages (sebaceous glands, hair follicles). Ki67 immunoexpression was detected in 19 cases (61.29%) within the control group and in 100% of AK lesions prior to treatment.

**Figure 2:**
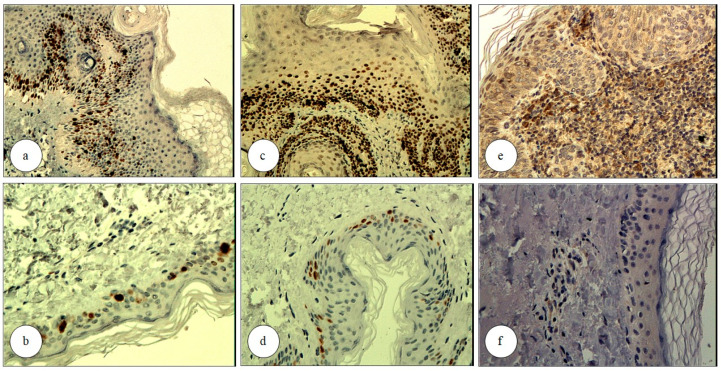
Immunoexpression of selected markers in an actinic keratosis (AK) lesion before and after photodynamic therapy (PDT); Ki67 immunostaining of an AK lesion before (**a**) and after (**b**) PDT, demonstrating a reduction in Ki67-positive cells following treatment (Immunohistochemistry (IHC), nuclear staining, magnification ×100 and ×200, respectively); p53 immunostaining of an AK lesion before (**c**) and after (**d**) PDT, demonstrating a reduction in p53-positive cells post-treatment (IHC, strong nuclear and weak cytoplasmic staining; magnification ×150 and ×200, respectively); immunoexpression of COX-2 in AK before (**e**) and after (**f**) PDT, showing a reduction in COX-2 expression post-treatment (IHC, cytoplasmic staining; magnification ×150 and ×200, respectively).

Following treatment, Ki67 immunoexpression was negative in three cases (9.68%) and reduced in all cases (100%) (Figure 2). According to the evaluation criteria, samples with fewer than 5% Ki67+ and p53+ cells per HPF were classified as negative (Table 3).

**Figure 3:**
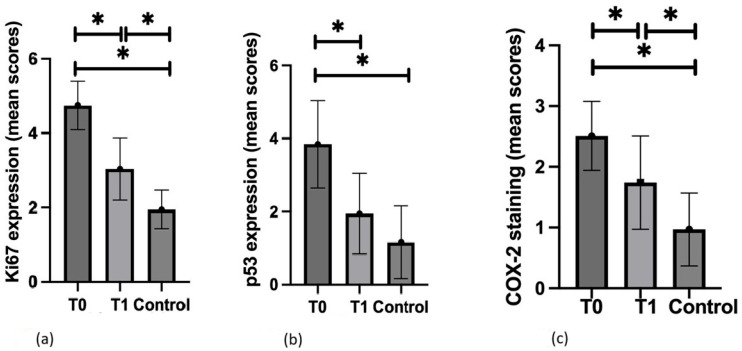
Statistical evaluation of selected marker expression within actinic keratosis (AK) lesions before (T0) and after (T1) the photodynamic therapy (PDT) and apparently healthy skin (Control)—(**a**) Ki67; (**b**) p53; (**c**) COX-2.; * ***p* < 0.05**.

Regarding statistical analysis, Ki67 epidermal immunopositivity in AK lesions significantly decreased post-treatment (2.70 ± 1.16 vs. 1.51 ± 1.02; *p* < 0.05), further supporting the antiproliferative effect of PDT (Figure 3a). Similarly, the extent of Ki67+ cells in the epidermis of AK lesions also showed a significant reduction (2.00 ± 0.51 vs. 1.41 ± 0.62; *p* < 0.05). The sum of both immunoexpression and the extent of Ki67-possitive cells decreased significantly (Figure 3a).

### 3.3. p53

Regarding p53+ cells (nuclear expression), 6 cases (19.35%) in the control group showed positivity, while 23 cases (74.19%) of AK lesions were positive prior to treatment. Following PDT, 17 cases (54.84%) became completely negative, and p53 expression decreased in all cases compared to pre-treatment levels (Figure 2).

The reduction in p53 immunoexpression following PDT was statistically significant (2.38 ± 1.68 vs. 0.87 ± 1.20; *p* < 0.05) (Figure 3b). However, changes in the extent of p53+ cells within the epidermis of AK lesions were not statistically significant (1.45 ± 1.02 vs. 1.06 ± 0.89; *p* = 0.12). The reduction in p53 expression following PDT was comparable to the expression of p53 in apparently healthy skin (0.87 ± 1.20 vs. 0.61 ± 1.68; *p* > 0.05). The sum of both the immunoexpression and the extent of p53-possitive cells decreased significantly (Figure 3b).

### 3.4. COX-2

COX-2 cytoplasmic immunoexpression (0.97 ± 0.60) was positive in 17 cases (54.84%) in the control group and in all AK lesions (100%) prior to treatment. Following PDT, COX-2 immunoexpression became negative in two cases (6.45%) and decreased in the remaining cases (Figure 2).

Overall, COX-2 staining significantly decreased in treated skin compared to baseline (2.51 ± 0.57 vs. 1.74 ± 0.77; *p* < 0.05), further supporting the anti-inflammatory effect of PDT (Figure 3c).

Additionally, post-treatment analysis revealed a positive correlation between the reduction in Ki67, p53, and COX-2 immunoexpression and a decrease in AK severity (both according to Olsen grade and dermatoscopic grade).

## 4. Discussion

AKs are common pre-neoplastic skin lesions that can evolve into NMSCs, primarily cSCC. Photodynamic therapy (PDT) is an effective field-directed treatment for AKs, offering the advantage of targeting the entire “field cancerization” (FC). Early intervention is crucial to preventing malignant transformation, particularly into cSCC. In mild to moderate AK cases, a single session of c-PDT (using 16% MAL, 8 mg of 5-ALA plaster, or 75 mg/g of 5-ALA) is typically recommended, with a follow-up evaluation after three months [44].

In cases of severe AK lesions, treatment becomes more complex, as no standardized guidelines are currently available. In our study protocol, we planned to perform three PDT sessions at four-week intervals, with the final follow-up visit scheduled eight weeks after the last PDT session. Every four weeks, prior to each PDT session, AK lesions were reassessed through both clinical and dermatoscopic examinations to monitor treatment response and disease progression.

The clinical, dermatoscopic, and immunohistochemical findings in our study, demonstrating a decrease in proliferation markers (Ki67 and p53) and the inflammation marker (COX-2), confirm the effectiveness of PDT in managing AKs. These results suggest that PDT may also help delay, or potentially inhibit, the carcinogenic process in the epidermis.

Several studies have already demonstrated a decrease in Ki67 and p53 expression following therapy for AK, including PDT. However, their findings suggest that a single or even two PDT sessions may not be sufficient for optimal efficacy [45,46,47,48].

For example, Campione et al. demonstrated that Ki67 expression decreased following AK treatment with three different modalities: 0.8% piroxicam cream, PDT, and ingenol mebutate gel. This further supports the efficacy of these therapies in reducing cellular proliferation in AK lesions [45]. In their study, PDT treatment was administered using a protocol consisting of two treatment cycles, each spaced four weeks apart, with a final reassessment conducted at 16 weeks from baseline. This protocol aligns closely with our study design, though we implemented three PDT sessions at four-week intervals, with final evaluation eight weeks post-treatment.

The study by Gellén et al., which involved 11 patients with multiple AKs on the scalp, face, hands, or forearms, treated with conventional 5-aminolevulinic acid PDT (c-5ALA-PDT) and Er:YAG laser-assisted 5-aminolevulinic acid PDT (5ALA-PDT) in a split-site manner, confirmed a significant decrease in the number of Ki67- and p53-positive cells (with analysis conducted before the PDT session, and at 48 h and 3 months after treatment). However, the abnormal cells were not completely eliminated after treatment, suggesting the need for multiple PDT sessions [46].

Furthermore, Abdalla et al. demonstrated that dl-PDT also reduces atypia and controls Ki67 expression, decreasing the proliferation of atypical cells and suggesting its potential in controlling carcinogenesis. According to their study, after dl-PDT, Ki67 expression decreased or remained unchanged in both biopsied areas (AK lesions and FC before and after dl-PDT). However, similar to their findings, residual Ki67 positivity remained in some cases, reinforcing the notion that multiple PDT sessions may be required to achieve sustained lesion clearance and reduce recurrence risk [47].

Bagazgoitia et al. also demonstrated that c-PDT with methyl aminolevulinic acid (MAL-cPDT) reduced Ki67 expression in 22 AK cases, indicating a decrease in the proliferative activity of the epidermis. They found that while Ki67 is normally expressed in a healthy epidermis, it is more intensely expressed in AKs. Ki67 was overexpressed in 20 of 22 (91%) cases, and its expression was reduced after PDT in 17 of 22 (77%) cases (*p* < 0.0001), with levels similar to those in control skin. Meanwhile, p53 expression significantly decreased in 11 of 22 (50%) AK cases (*p* < 0.002) but remained stable in the other 11 (50%). However, a complete disappearance of p53, which should not be expressed in healthy skin, was observed in only one case (5%). These results suggest that PDT is effective in reducing these markers, but a single treatment may not be sufficient to fully eliminate actinic damage [48].

The optimal number of PDT sessions required to completely eliminate carcinogenic changes remains undetermined. Given reports that one or two sessions may often be insufficient, our study protocol employed three PDT sessions spaced four weeks apart to maximize therapeutic efficacy. This regimen resulted in complete clinical remission of AK lesions in 17 patients (54.8%), while the remaining 14 patients (45.2%) achieved a partial response, with all residual lesions regressing to Olsen grade 1 (mild). Importantly, no patient had lesions of Olsen grade 2 or 3 after therapy, indicating that all remaining lesions were of the least severe grade. These findings support the need for multiple treatment cycles to optimize outcomes, but also illustrate that even a three-session regimen can leave residual disease in a substantial proportion of patients. The presence of persistent grade 1 lesions in nearly half of the treated patients raises critical questions about the completeness of the therapeutic response and the long-term prognosis. It is unclear whether these residual lesions represent an incomplete treatment response or early recurrences of AK. Distinguishing between these scenarios is clinically important: if the lesions reflect incomplete clearance, additional PDT sessions or adjunctive therapies might be required to achieve full lesion eradication, whereas if they are early recurrences, their presence could indicate underlying FC (persistent subclinical dysplasia in the surrounding skin) and thus warrant more vigilant surveillance or more aggressive field-directed treatment. Regardless of the underlying cause, a comprehensive long-term follow-up is warranted for all patients with a history of AK, not only those with residual lesions after therapy. Such follow-up should include regular clinical and dermoscopic examinations to monitor for new or recurrent lesions and to ensure early detection of any progression to cSCC. Further research is needed to evaluate the long-term efficacy of repeated PDT sessions and to clarify their role in preventing AK recurrence and progression to cSCC.

Similarly to the studies mentioned above, our study also confirmed that the expression of Ki67 and p53 in the epidermis of AK lesions persisted after treatment (28/31 subjects, 90.32% for Ki67; 14/31, 45.16% for p53). We observed that in six cases (6/31, 9.35%), the immunoexpression of Ki67 remained unchanged after treatment, and in one case (1/31, 3.22%), it increased. These findings suggest that intense keratinocyte proliferation is still occurring in these lesions. However, it remains unclear whether this indicates a recurrence of AK lesions or an ongoing epidermal regeneration process following PDT.

Notably, p53 expression remained unchanged in only one subject (1/31, 3.22%), while it decreased in all other cases post-treatment. Our findings suggest that p53 may be a more specific biomarker for monitoring AK treatment compared to Ki67.

However, this conclusion remains inconclusive, as previous studies have reported a less pronounced reduction in p53 expression following PDT compared to Ki67. Further research is needed to clarify the role of p53 in AK regression and its potential as a treatment response indicator. Interestingly, our findings differ from some of the previously published data, as we observed a significant decrease in p53 immunoexpression post-PDT [45,46,47,48]. This suggests that PDT may exert its therapeutic effect not only by reducing proliferation but also by modulating key regulatory pathways involved in cell cycle control and apoptosis. A possible explanation is that while Ki67 expression does not always decrease uniformly, likely due to ongoing regenerative processes in the epidermis, p53 downregulation may indicate the effective suppression of DNA damage responses and a shift toward normal keratinocyte turnover.

On the other hand, it is important to acknowledge the limitations of our study, particularly the relatively small sample size. This could impact the generalizability of our findings and may partially account for discrepancies with previous reports. Further large-scale, long-term studies are necessary to determine the precise molecular mechanisms underlying PDT’s effects on p53 and Ki67 expression and to establish whether p53 downregulation serves as a reliable biomarker of treatment efficacy.

Overall, a sustained reduction in Ki67 expression following PDT for AK indicates a decreased proliferative fraction of keratinocytes, consistent with therapeutic efficacy and a reduced likelihood of progression to iSCC. In contrast, persistently elevated Ki67 levels post-treatment may be predictive of subclinical residual disease and increased recurrence risk. Similarly, p53 overexpression, particularly if it reflects the accumulation of mutant, non-functional p53 protein, correlates with genomic instability and neoplastic progression [30]. A post-treatment decrease in p53 expression is considered indicative of a reduced mutational burden and normalization of DNA repair mechanisms. By contrast, persistent p53 overexpression in the absence of clinical or histological improvement suggests residual dysplasia or ongoing malignant potential. Regarding our study, for Ki67, we can propose a post-treatment positivity in <15% of keratinocyte nuclei (which means a low proliferative index) as an effective lesion clearance. Similarly, a post-treatment p53-positive nuclear fraction below 5% is considered a strong predictor of diminished mutant clones and restored DNA repair function, consistent with a favorable prognosis.

Subsequently, COX-2 overexpression is well documented in AK as well as in NMSC lesions, with multiple studies supporting its role in skin carcinogenesis. A study by Kim et al. confirmed elevated COX-2 and p53 expression in AK, SCC, BCC, and Bowen disease (BD). However, according to the study results, while both markers are implicated in tumor progression, their correlation in skin tumors was not statistically significant (*p* > 0.05). This suggests that while COX-2 and p53 may contribute independently to carcinogenesis, their interplay remains complex and requires further investigation [22].

Similarly, Wu et al. reported significantly higher COX-2 expression in SCC, BD, and AK compared to seborrheic keratosis (*p* < 0.01), reinforcing its role in skin carcinogenesis. Amirnia et al. further confirmed elevated COX-2 levels in both malignant (SCC, BCC) and pre-malignant (BD, AK) lesions, suggesting that COX-2 could serve as a molecular target for treating various skin tumors [49,50].

Further supporting these findings, Adamska et al. conducted an immunohistochemical analysis of COX-2 expression in 94 AK skin samples. While COX-2 levels were slightly higher in KIN2 (lesions with keratinocytic atypia in the lower two-thirds of the epidermis) AK lesions compared to KIN1 (lesions with keratinocytic atypia in the lower third of the epidermis) (with no expression in KIN3, lesions with atypical cells throughout the epidermis), no statistically significant correlation was found between COX-2 intensity and AK stage. Additionally, COX-2 expression appeared independent of patient age, sex, or skin phenotype, suggesting that its role in AK may be more complex than a simple marker of disease progression [51]. Furthermore, our data also showed no significant association between a reduction in COX-2 levels (as well as in Ki67 and p53) and patient age or sex. However, we observed a better clinical response in younger patients (younger than 72.4 years old). This observation may be attributed to a more efficient immune response and accelerated wound healing capacity typically observed in younger skin. In our analysis, the clinical efficacy of PDT did not demonstrate a statistically significant association with patient sex. Both male and female participants exhibited comparable rates of lesion regression and biomarker normalization following therapy. Nonetheless, our analysis is limited by the relatively small number of female participants in the study group, which may reduce the statistical power to detect subtle sex-based differences in response. As a result, while our data suggest the comparable efficacy of PDT across sexes, this conclusion should be interpreted with caution and validated in larger, sex-balanced cohorts.

One of the most critical aspects of COX-2 involvement in AK pathogenesis is its inhibition by NSAIDs, such as diclofenac, which remain a widely used topical treatment for AK. This makes it particularly intriguing to investigate whether PDT influences COX-2 expression in AK lesions. To the best of our knowledge, no prior study has examined this phenomenon. Our study is the first to assess the impact of PDT on COX-2 expression in AK. Our findings demonstrate a significant reduction in COX-2 immunoexpression following PDT (Figure 3c). However, in two cases, COX-2 expression unexpectedly increased post-treatment, while in nine cases it remained unchanged (2/31, 6.45% and 9/31, 29.03%, respectively). We hypothesize that this may be attributed to local regenerative inflammation induced by reactive oxygen species (ROS) during PDT. This observation aligns with the concept presented by Karampinis et al., who describe a cyclical interaction between oxidative stress, inflammation, and immune modulation in non-melanoma skin cancer. PDT-induced ROS may disrupt local immune tolerance and initiate immune activation, by facilitating the release of damage-associated molecular patterns (DAMPs), thereby promoting antigen presentation and the recruitment of immune effector cells. Concurrently, ROS-induced alterations in the tumor microenvironment may stimulate the production of immunoregulatory cytokines and foster phenotypic plasticity in resident immune cells, thereby contributing to sustained immunosurveillance. On the other hand, if oxidative-inflammatory signaling persists, it may hinder complete resolution. These findings suggest that, while PDT offers both cytotoxic and immunomodulatory benefits, certain cases may require combination therapies to fully break the ROS–inflammation cycle and ensure long-term lesion clearance [52].

Summing up, PDT led to a marked reduction in COX-2 expression in treated AK lesions, indicating a shift toward an anti-inflammatory state and a more normalized tumor microenvironment. COX-2 overexpression is linked to UV-induced inflammation and tumor progression in skin, so its suppression suggests that the lesion is less conducive to carcinogenesis. Notably, baseline COX-2 levels do not strictly correlate with AK severity: Adamska et al. found no linear relationship between COX-2 immunoreactivity and histological grade, with advanced lesions (KIN III) sometimes showing no COX-2 at all [51]. Thus, while an absence or minimal COX-2 immunoexpression intensity post-treatment may signify therapeutic effect, this finding should be interpreted with caution, as a regenerating epithelium can also exhibit low COX-2 in the early healing phase [50,51]. Overall, a concurrent reduction in COX-2, Ki67, and p53 provides the most reliable indication of treatment efficacy and a lower risk of recurrence. This combined biomarker response reflects decreased inflammation (COX-2), reduced cellular proliferation (Ki67), and the clearance of dysplastic or mutated cell clones (p53). In fact, effective PDT is known to downregulate proliferation markers like Ki67 and oncogenic stress markers like p53 in AK, correlating with reduced growth and increased apoptosis of atypical keratinocytes [53]. The present findings align with this, suggesting that multi-faceted marker suppression is a hallmark of successful lesion regression. Establishing standardized cutoff values for these biomarkers enhances their potential utility in routine post-treatment surveillance and risk stratification protocols; therefore, future prospective studies are essential.

Interestingly, similar biomarker improvements are observed not only in PDT but also in other field therapies for AK. For example, topical diclofenac 3% (a COX-2 inhibitor) significantly decreases COX-2 expression and T-cell infiltration (CD3+ and CD8+ cells) in AK, along with a clear reduction in angiogenic factor CD31. While its effect on proliferation markers did not reach significance, there were notable downward trends in Ki67 levels but no significant change in p53 levels after the treatment [54]. Ingenol mebutate gel likewise induces substantial declines in proliferative and atypical cell markers: in one study, Ki67 levels dropped significantly (*p* = 0.015) and the stem cell-associated marker p63 was markedly reduced (*p* = 0.002) after treatment. These effects reflect both cytotoxic and immunomodulatory actions of ingenol, including the induction of rapid cell death and immune cell recruitment [55]. Finally, Campione et al. further demonstrated that 0.8% piroxicam (another topical NSAID) can also be effective in reducing Ki67 expression (*p* < 0.01) [45]. Moreover, a recent randomized trial of topical colchicine 0.5% reported a significant 15% decrease in Ki67 and a significant 27% reduction in p53-positive cells in treated field areas. Given colchicine’s antimitotic action via microtubule inhibition, the downregulation of Ki67 aligns with its known antiproliferative effects. The substantial reduction in p53 may suggest the effective clearance of dysplastic cells with DNA damage, leading to the normalization of apoptotic regulatory pathways [56].

In the end, the final important aspect involved the interventions undertaken by our team to reduce patient discomfort (primarily pain and burning sensations in the treatment area) reported during irradiation. Among the 16 patients who experienced AEs, we introduced modifications to the PDT parameters during irradiation in 14 cases. In 12 of these patients, the adjustments had no observable impact on the clinical outcome or therapeutic efficacy. However, in the remaining two individuals, a significant reduction in the number or extent of skin lesions was not achieved following treatment, suggesting that the modified protocol may have contributed to the suboptimal therapeutic effect in these particular cases. This observation should be taken into account when considering similar adjustments during treatment. On the other hand, these findings also emphasize that the procedure may inherently involve a degree of patient discomfort, and that individuals with a lower pain threshold may require tailored modifications to ensure tolerability without compromising efficacy.

## 5. Conclusions

This study evaluates the effectiveness of PDT in treating AK by analyzing clinical, dermatoscopic, and immunohistochemical changes, particularly in the expression of proliferation (Ki67, p53) and inflammation (COX-2) biomarkers. Thirty-one patients underwent three PDT sessions at four-week intervals, with final follow-up at eight weeks post-treatment. Clinical and dermatoscopic assessments revealed significant lesion regression, with 54.8% of patients achieving complete resolution. Immunohistochemical analysis demonstrated a significant reduction in Ki67, p53, and COX-2 immunoexpression post-treatment, confirming PDT’s antiproliferative and anti-inflammatory effects.

Ki67 and p53 can potentially serve as biomarkers for monitoring and prognosis in the treatment of AK, particularly in assessing response to PDT and evaluating the risk of progression to SCC. A decrease in Ki67 immunoexpression post-PDT suggests the effective suppression of cell proliferation and therapeutic response whereas a decrease in p53 immunoexpression decreased DNA-damaged or transformed cells. Persistent high levels of Ki67 may indicate incomplete lesion clearance or potential for recurrence, whereas, some p53 mutations are stable and persistent, making interpretation more complex. High and persistent p53 expression may indicate higher risk of progression to invasive SCC.

Notably, this study is the first to examine the impact of PDT on COX-2 expression. COX-2 levels typically decrease after successful PDT, correlating with reduced inflammatory and neoplastic activity. Sustained or increased COX-2 expression post-treatment could signal ongoing ROS-driven local regenerative inflammation during PDT. Eventually, COX-2 can also be potentially considered as a biomarker for monitoring and prognosis in the treatment of AK.

Ultimately, the results support PDT’s role in delaying carcinogenesis and improving cosmetic outcomes. However, persistent expression of these markers in some cases suggests that additional PDT sessions or combination therapies may be necessary for complete lesion clearance. Further studies are needed to establish optimal treatment protocols, particularly regarding COX-2 modulation and its implications in AK progression and recurrence.

## Figures and Tables

**Table 1:** Inclusion and exclusion criteria for the study.

**Inclusion Criteria**	
1	Signed informed consent for participation in the study.
2	Age ≥ 18 years.
3	Presence of a primary AK lesion with characteristic morphology, previously untreated.
4	Good general health, with no clinically significant conditions as assessed by the physician.
5	Ability to comprehend study-related information, adhere to protocol requirements, follow medical recommendations, and attend scheduled visits.
**Exclusion Criteria**	
1	Hypersensitivity to UV or blue light.
2	Age < 18 years.
3	Pregnant or breastfeeding women, or those planning pregnancy during the study.
4	Participation in another clinical trial within 30 days prior to study initiation.
5	Inability or unwillingness to comply with study requirements.
6	Presence of acute inflammatory disease at the time of examination, with body temperature > 38 °C.
7	Active malignancy or history of cancer, including skin cancers (SCC, BCC, MM).
8	Autoimmune diseases, including Hashimoto’s thyroiditis, Graves’ disease, type 1 diabetes, rheumatoid arthritis, celiac disease, Crohn’s disease, systemic lupus erythematosus, dermatomyositis, polymyositis, ankylosing spondylitis, polymyalgia rheumatica, multiple sclerosis, Sjögren’s syndrome, and vitiligo.
9	Impaired wound healing.
10	Blood clotting disorders.
11	Chronic or intermittent use of NSAIDs during the study period.
12	Long-term anticoagulant therapy.
13	Long-term use of medications with known phototoxic effects.
14	Long-term immunosuppressive, immunomodulatory, or systemic corticosteroid therapy.
15	Planned hospitalization or surgical procedures during the study period.
16	Diastolic blood pressure > 95 mmHg or <65 mmHg.
17	Congenital or acquired immunodeficiency disorders.
18	Genophotodermatoses associated with increased skin cancer risk, such as xeroderma pigmentosum, Cockayne syndrome, and Bloom syndrome.
19	Renal dysfunction.
20	Psychiatric disorders with psychotic symptoms or major depressive disorder.
21	Substance abuse or dependence (alcohol, prescription medications, or illicit drugs) within the last 12 months.
22	Use of tanning beds or artificial UV exposure.

**Abbreviations:** AK, actinic keratosis; UV, ultraviolet; SCC, squamous cell carcinoma; BCC, basal cell carcinoma; MM, malignant melanoma; NSAIDs, non-steroidal anti-inflammatory drugs.

**Table 2:** Assessment of the severity of actinic keratosis (AK) in the Clinical Classification (Olsen’s) and Dermatoscopic Severity Scale proposed by Zalaudek et al. [35] Given the FC and the broader area affected by varying grades of AKs, we proposed the modified version of the Dermatoscopic Severity Scale and we also introduced intermediate values, such as I/II and II/III, to better represent these transitional stages.

**Grade**	**Olsen’s Clasification System**
1	lesions are slightly palpable, with the AK being more felt than visible
2	lesions are moderately thick, easily felt, and visible
3	lesions are very thick, hyperkeratotic, and/or clearly noticeable as AK
**Grade**	**Dermatoscopic Severity Scale [35] with Our Modification**
I	red pseudo-network pattern with discrete white scales
I/II	features of I and II grade
II	an erythematous background, the so-called “strawberry pattern”
II/III	features of II and III grade
III	enlarged follicular openings with keratotic plugs over a scaly, white-to-yellow background

**Table 3:** Biomarker expression analysis.

The expression of Ki67 and p53 (nuclear staining) was graded based on the mean percentage of immunopositive cells per high-power field (HPF):	
**Score**	**Immunopositive Cells (%)**
0	<5%
1	6–15%
2	16–30%
3	31–50%
4	>50%
For Ki67 and p53, the extent of immunopositive cells in the epidermis was graded as follows:	
**Score**	**Extent of Immunopositive Cells in the Epidermis**
0	No immunopositive cells
1	<1/3 of the lower epidermal/lesion level
2	1/3–2/3 of the epidermal/lesion level
3	2/3 to the entire epidermis
The expression of COX-2 (cytoplasmic staining) was graded based on mean immunoexpression intensity:	
**Score**	**Immunoexpression Intensity**
0	Not detectable
1	Mild
2	Moderate
3	Strong

## Data Availability

The data presented in this study are available on request from the corresponding author.

## Abbreviations

The following abbreviations are used in this manuscript:

AK	Actinic keratosis
cSCC	Cutaneous squamous cell carcinoma
UV	Ultraviolet
iSCC	Invasive squamous cell carcinoma
BCC	Basal cell carcinoma
FC	Field cancerization
PDT	Photodynamic therapy
c-PDT	Conventional PDT
dl-PDT	Daylight PDT
ALA	5-aminolevulinic acid
MAL	Methyl aminolevulinate
COX-2	Cyclooxygenase-2
PGE2	Prostaglandin E2
NMSC	Non-melanoma skin cancer
MM	Malignant melanoma
NSAIDs	Nonsteroidal anti-inflammatory drugs
HPF	High-power fields
AEs	Adverse events
c-5-ALAPDT	5-aminolevulinic acid PDT
MAL-cPDT	c-PDT with methyl aminolevulinic acid
BD	Bowen disease
ROS	Reactive oxygen species
KIN	Keratinocytic intraepithelial neoplasia

## References

[1] Ferrándiz C., Plazas M.J., Sabaté M., Palomino R., EPIQA Study Group (2016). Prevalence of actinic keratosis among dermatology outpatients in Spain. Actas Dermo-Sifiliogr..

[2] Yaldiz M. (2019). Prevalence of actinic keratosis in patients attending the dermatology outpatient clinic. Medicine.

[3] Kandolf L., Peris K., Malvehy J., Mosterd K., Heppt M.V., Fargnoli M.C., Berking C., Arenberger P., Bylaite-Bučinskiene M., Del Marmol V. (2024). European consensus-based interdisciplinary guideline for diagnosis, treatment and prevention of actinic keratoses, epithelial UV-induced dysplasia and field cancerization on behalf of European Association of Dermato-Oncology, European Dermatology Forum, European Academy of Dermatology and Venereology and Union of Medical Specialists (Union Européenne des Médecins Spécialistes). J. Eur. Acad. Dermatol. Venereol..

[4] Heppt M.V., Leiter U., Steeb T., Amaral T., Bauer A., Becker J.C., Breitbart E., Breuninger H., Diepgen T., Dirschka T. (2020). S3 guideline for actinic keratosis and cutaneous squamous cell carcinoma—Short version, part 1: Diagnosis, interventions for actinic keratoses, care structures and quality-of-care indicators. J. Dtsch. Dermatol. Ges..

[5] Glogau R.G. (2000). The risk of progression to invasive disease. J. Am. Acad. Dermatol..

[6] Balcere A., Konrāde-Jilmaza L., Pauliņa L.A., Čēma I., Krūmiņa A. (2022). Clinical Characteristics of Actinic Keratosis Associated with the Risk of Progression to Invasive Squamous Cell Carcinoma: A Systematic Review. J. Clin. Med..

[7] Wehner M.R., Linos E., Parvataneni R., Stuart S.E., Boscardin W.J., Chren M.M. (2015). Timing of subsequent new tumors in patients who present with basal cell carcinoma or cutaneous squamous cell carcinoma. JAMA Dermatol..

[8] Que S.K.T., Zwald F.O., Schmults C.D. (2018). Cutaneous squamous cell carcinoma: Incidence, risk factors, diagnosis, and staging. J. Am. Acad. Dermatol..

[9] Cohen J.L. (2010). Actinic keratosis treatment as a key component of preventive strategies for nonmelanoma skin cancer. J. Clin. Aesthet. Dermatol..

[10] Dirschka T., Gupta G., Micali G., Stockfleth E., Basset-Séguin N., Del Marmol V., Dummer R., Jemec G.B.E., Malvehy J., Peris K. (2017). Real-world approach to actinic keratosis management: Practical treatment algorithm for office-based dermatology. J. Dermatolog. Treat..

[11] Willenbrink T.J., Ruiz E.S., Cornejo C.M., Schmults C.D., Arron S.T., Jambusaria-Pahlajani A. (2020). Field cancerization: Definition, epidemiology, risk factors, and outcomes. J. Am. Acad. Dermatol..

[12] Eisen D.B., Asgari M.M., Bennett D.D., Connolly S.M., Dellavalle R.P., Freeman E.E., Goldenberg G., Leffell D.J., Peschin S., Sligh J.E. (2021). Guidelines of care for the management of actinic keratosis. J. Am. Acad. Dermatol..

[13] Dirschka T., Radny P., Dominicus R., Mensing H., Brüning H., Jenne L., Karl L., Sebastian M., Oster-Schmidt C., Klövekorn W. (2012). Photodynamic therapy with BF-200 ALA for the treatment of actinic keratosis: Results of a multicentre, randomized, observer-blind phase III study in comparison with a registered methyl-5-aminolaevulinate cream and placebo. Br. J. Dermatol..

[14] Dirschka T., Radny P., Dominicus R., Mensing H., Brüning H., Jenne L., Karl L., Sebastian M., Oster-Schmidt C., Klövekorn W. (2013). Long-term (6 and 12 months) follow-up of two prospective, randomized, controlled phase III trials of photodynamic therapy with BF-200 ALA and methyl aminolaevulinate for the treatment of actinic keratosis. Br. J. Dermatol..

[15] Reinhold U., Dirschka T., Ostendorf R., Aschoff R., Berking C., Philipp-Dormston W., Hahn S., Lau K., Jäger A., Schmitz B. (2016). A randomized, double-blind, phase III, multicentre study to evaluate the safety and efficacy of BF-200 ALA (Ameluz(®)) vs. placebo in the field-directed treatment of mild-to-moderate actinic keratosis with photodynamic therapy (PDT) when using the BF-RhodoLED(®) lamp. Br. J. Dermatol..

[16] Szeimies R.-M., Radny P., Sebastian M., Borrosch F., Dirschka T., Krähn-Senftleben G., Reich K., Pabst G., Voss D., Foguet M. (2010). Photodynamic therapy with BF-200 ALA for the treatment of actinic keratosis: Results of a prospective, randomized, double-blind, placebo-controlled phase III study. Br. J. Dermatol..

[17] Costa C., Scalvenzi M., Ayala F., Fabbrocini G., Monfrecola G. (2015). How to treat actinic keratosis? An update. J. Dermatol. Case Rep..

[18] Miola A.C., Castilho M.A., Schmitt J.V., Marques M.E.A., Miot H.A. (2019). Contribution to characterization of skin field cancerization activity: Morphometric, chromatin texture, proliferation, and apoptosis aspects. An. Bras. Dermatol..

[19] Hollstein M., Sidransky D., Vogelstein B., Harris C.C. (1991). p53 mutations in human cancers. Science.

[20] Carpenter P.M., Linden K.G., McLaren C.E., Li K.-T., Arain S., Barr R.J., Hite P., Sun J.D., Meyskens F.L. (2004). Nuclear morphometry and molecular biomarkers of actinic keratosis, sun-damaged, and nonexposed skin. Cancer Epidemiol. Biomarkers Prev..

[21] Stratigos A., Kapranos N., Petrakou E., Anastasiadou A., Pagouni A., Christofidou E., Petridis A., Papadopoulos O., Kokka E., Antoniou C. (2005). Immunophenotypic analysis of the p53 gene in non-melanoma skin cancer and correlation with apoptosis and cell proliferation. J. Eur. Acad. Dermatol. Venereol..

[22] Kim K.H., Park E.J., Seo Y.J., Cho H.S., Kim C.W., Kim K.J., Park H.R. (2006). Immunohistochemical study of cyclooxygenase-2 and p53 expression in skin tumors. J. Dermatol..

[23] Piipponen M., Riihilä P., Nissinen L., Kähäri V.M. (2021). The Role of p53 in Progression of Cutaneous Squamous Cell Carcinoma. Cancers.

[24] Yilmaz A.S., Ozer H.G., Gillespie J.L., Allain D.C., Bs M.N.B., Furlan K.C., Castro L.T.F., Peters S.B., Nagarajan P., Kang S.Y. (2017). Differential mutation frequencies in metastatic cutaneous squamous cell carcinomas versus primary tumors. Cancer.

[25] McGregor J.M., Yu C.C., Dublin E.A., Levison D.A., MacDonald D.M. (1992). Aberrant expression of p53 tumour-suppressor protein in non-melanoma skin cancer. Br. J. Dermatol..

[26] Taguchi M., Watanabe S., Yashima K., Murakami Y., Sekiya T., Ikeda S. (1994). Aberrations of the tumor suppressor p53 gene and p53 protein in solar keratosis in human skin. J. Investig. Dermatol..

[27] Nelson M.A., Einspahr J.G., Alberts D.S., Balfour C.A., Wymer J.A., Welch K.L., Salasche S.J., Bangert J.L., Grogan T.M., Bozzo P.O. (1994). Analysis of the p53 gene in human precancerous actinic keratosis lesions and squamous cell cancers. Cancer Lett..

[28] Hedberg M.L., Berry C.T., Moshiri A.S., Xiang Y., Yeh C.J., Attilasoy C., Capell B.C., Seykora J.T. (2022). Molecular Mechanisms of Cutaneous Squamous Cell Carcinoma. Int. J. Mol. Sci..

[29] Prior S.L., Griffiths A.P., Lewis P.D. (2009). A study of mitochondrial DNA D-loop mutations and p53 status in nonmelanoma skin cancer. Br. J. Dermatol..

[30] Balcere A., Sperga M., Čēma I., Lauskis G., Zolovs M., Kupfere M.R., Krūmiņa A. (2023). Expression of p53, p63, p16, Ki67, Cyclin D, Bcl-2, and CD31 Markers in Actinic Keratosis, In Situ Squamous Cell Carcinoma and Normal Sun-Exposed Skin of Elderly Patients. J. Clin. Med..

[31] Berman B., Cockerell C.J. (2013). Pathobiology of actinic keratosis: Ultraviolet-dependent keratinocyte proliferation. J. Am. Acad. Dermatol..

[32] Wang D., Dubois R.N. (2010). Eicosanoids and cancer. Nat Rev Cancer..

[33] Elmets C.A., Viner J.L., Pentland A.P., Cantrell W., Lin H.-Y., Bailey H., Kang S., Linden K.G., Heffernan M., Duvic M. (2010). Chemoprevention of nonmelanoma skin cancer with celecoxib: A randomized, double-blind, placebo-controlled trial. J. Natl. Cancer Inst..

[34] Zalaudek I., Giacomel J., Argenziano G., Hofmann-Wellenhof R., Micantonio T., Di Stefani A., Oliviero M., Rabinovitz H., Soyer H., Peris K. (2006). Dermoscopy of facial nonpigmented actinic keratosis. Br. J. Dermatol..

[35] Zalaudek I., Piana S., Moscarella E., Longo C., Zendri E., Castagnetti F., Pellacani G., Lallas A., Argenziano G. (2014). Morphologic grading and treatment of facial actinic keratosis. Clin. Dermatol..

[36] Martín J.M., Bella-Navarro R., Jordá E. (2012). Vascularización en dermatoscopia [Vascular patterns in dermoscopy]. Actas Dermo-Sifiliogr..

[37] Olsen E.A., Abernethy M.L., Kulp-Shorten C., Callen J.P., Glazer S.D., Huntley A., McCray M., Monroe A.B., Tschen E., Wolf J.E. (1991). A double-blind, vehicle-controlled study evaluating masoprocol cream in the treatment of actinic keratoses on the head and neck. J. Am. Acad. Dermatol..

[38] Zalaudek I., Giacomel J., Schmid K., Bondino S., Rosendahl C., Cavicchini S., Tourlaki A., Gasparini S., Bourne P., Keir J. (2012). Dermatoscopy of facial actinic keratosis, intraepidermal carcinoma, and invasive squamous cell carcinoma: A progression model. J. Am. Acad. Dermatol..

[39] Zalaudek I., Argenziano G. (2015). Dermoscopy of actinic keratosis, intraepidermal carcinoma and squamous cell carcinoma. Curr. Probl. Dermatol..

[40] Dianzani C., Conforti C., Giuffrida R., Corneli P., di Meo N., Farinazzo E., Moret A., Rizzi G.M., Zalaudek I. (2020). Current therapies for actinic keratosis. Int. J. Dermatol..

[41] Park M.Y., Sohn S., Lee E.S., Kim Y.C. (2010). Photorejuvenation induced by 5-aminolevulinic acid photodynamic therapy in patients with actinic keratosis: A histologic analysis. J. Am. Acad. Dermatol..

[42] Oyama J., Ramos-Milaré Á.C.F.H., Lera-Nonose D.S.S.L., Nesi-Reis V., Demarchi I.G., Aristides S.M.A., Teixeira J.J.V., Silveira T.G.V., Lonardoni M.V.C. (2020). Photodynamic therapy in wound healing in vivo: A systematic review. Photodiagnosis Photodyn. Ther..

[43] Nesi-Reis V., Lera-Nonose D.S.S.L., Oyama J., Silva-Lalucci M.P.P., Demarchi I.G., Aristides S.M.A., Teixeira J.J.V., Silveira T.G.V., Lonardoni M.V.C. (2018). Contribution of photodynamic therapy in wound healing: A systematic review. Photodiagnosis Photodyn. Ther..

[44] Morton C.A., Szeimies R.M., Basset-Seguin N., Calzavara-Pinton P., Gilaberte Y., Haedersdal M., Hofbauer G.F.L., Hunger R.E., Karrer S., Piaserico S. (2019). European Dermatology Forum guidelines on topical photodynamic therapy 2019 Part 1: Treatment delivery and established indications—Actinic keratoses, Bowen’s disease and basal cell carcinomas. J. Eur. Acad. Dermatol. Venereol..

[45] Campione E., Di Prete M., Di Raimondo C., Costanza G., Palumbo V., Garofalo V., Mazzilli S., Franceschini C., Dika E., Bianchi L. (2022). Topical Treatment of Actinic Keratosis and Metalloproteinase Expression: A Clinico-Pathological Retrospective Study. Int. J. Mol. Sci..

[46] Gellén E., Fidrus E., Janka E., Kollár S., Paragh G., Emri G., Remenyik É. (2019). 5-Aminolevulinic acid photodynamic therapy with and without Er:YAG laser for actinic keratosis: Changes in immune infiltration. Photodiagnosis Photodyn. Ther..

[47] Abdalla B.M.Z., Simas Pedreiro B., Garcia Morales A., Krutman Zveibil D., Paschoal F.M. (2022). Clinical, histopathological and immunohistochemical evaluation of daylight photodynamic therapy in the treatment of field cancerization: A study of 30 cases. J. Dermatolog. Treat..

[48] Bagazgoitia L., Cuevas Santos J., Juarranz A., Jaén P. (2011). Photodynamic therapy reduces the histological features of actinic damage and the expression of early oncogenic markers. Br. J. Dermatol..

[49] Wu Y., Liu H., Li J. (2007). Expression of p63 and cyclooxygenase-2 and their correlation in skin tumors. J. Huazhong Univ. Sci. Technol. Med. Sci..

[50] Amirnia M., Babaie-Ghazani A., Fakhrjou A., Khodaeiani E., Alikhah H., Naghavi-Behzad M., Zarrintan A. (2014). Immunohistochemical study of cyclooxygenase-2 in skin tumors. J. Dermatolog. Treat..

[51] Adamska K., Pawlaczyk M., Bowszyc-Dmochowska M., Gornowicz-Piotrowska J., Janicka-Jedyńska M., Fedorowicz T., Żaba R. (2018). Cyclooxygenase-2 expression in actinic keratosis. Postepy Dermatol. Alergol..

[52] Karampinis E., Koumaki D., Sgouros D., Nechalioti P.-M., Toli O., Pappa G., Papadakis M., Georgopoulou K.-E., Schulze-Roussaki A.-V., Kouretas D. (2025). Non-Melanoma Skin Cancer: Assessing the Systemic Burden of the Disease. Cancers.

[53] Naharro-Rodriguez J., Bacci S., Fernandez-Guarino M. (2024). Molecular Biomarkers in Cutaneous Photodynamic Therapy: A Comprehensive Review. Diagnostics.

[54] Maltusch A., Röwert-Huber J., Matthies C., Lange-Asschenfeldt S., Stockfleth E. (2011). Modes of action of diclofenac 3%/hyaluronic acid 2.5% in the treatment of actinic keratosis. J. Dtsch. Dermatol. Ges..

[55] Bobyr I., Campanati A., Consales V., Martina E., Molinelli E., Diotallevi F., Brisigotti V., Giangiacomi M., Ganzetti G., Giuliodori K. (2017). Ingenol mebutate in actinic keratosis: A clinical, videodermoscopic and immunohistochemical study. J. Eur. Acad. Dermatol. Venereol..

[56] Miola A.C., Ferreira E.R., Lima T.R.R., Schmitt J.V., Abbade L.P.F., Miot H.A. (2018). Effectiveness and safety of 0·5% colchicine cream vs. photodynamic therapy with methyl aminolaevulinate in the treatment of actinic keratosis and skin field cancerization of the forearms: A randomized controlled trial. Br. J. Dermatol..

